# Strengthening accountability for tuberculosis policy implementation in South Africa: perspectives from policymakers, civil society, and communities

**DOI:** 10.1186/s44263-024-00077-y

**Published:** 2024-07-17

**Authors:** Helene-Mari van der Westhuizen, Janet Giddy, Renier Coetzee, Goodman Makanda, Phumeza Tisile, Michelle Galloway, Siyavuya Bunyula, Ingrid Schoeman, Ruvandhi R. Nathavitharana

**Affiliations:** 1 TB Proof, Cape Town, South Africa; 2 Nuffield Department of Primary Care Health Sciences, Oxford University, Oxford, UK; 3 School of Public Health, University of the Western Cape, Cape Town, South Africa; 4 Beth Israel Deaconess Medical Center and Harvard Medical School, Boston, MA USA

**Keywords:** Tuberculosis, Accountability, Policy implementation, Advocacy, South Africa

## Abstract

**Background:**

Translating health policy into effective implementation is a core priority for responding effectively to the tuberculosis (TB) crisis. The national TB Recovery Plan was developed in response to the negative impact that the COVID-19 pandemic had on TB care in South Africa. We aimed to explore the implementation of the TB Recovery Plan and develop recommendations for strengthening accountability for policy implementation for this and future TB policies.

**Methods:**

We interviewed 24 participants working on or impacted by TB policy implementation in South Africa. This included perspectives from national, provincial, and local health department representatives, civil society, and community representatives. In-depth interviews were conducted in English and isiXhosa and we drew on reflexive thematic methods for analysis.

**Results:**

Participants felt that there was potential for COVID-19 innovations and urgency to influence TB policy development and implementation, including the use of data dashboards. Implementation of the TB Recovery Plan predominantly used a top-down approach to implementation (cascading from national policy to local implementers) but experienced bottlenecks at provincial level. Recommendations for closing the TB policy-implementation gap included using phased implementation and enhancing provincial-level accountability. Civil society organisations were concerned about the lack of provincial implementation data which impeded advocacy for improved accountability and inadequate resourcing for implementation. Community health workers were viewed as key to implementation but were not engaged in the policy development process and were often not aware of new TB policies. At local level, there were also opportunities to strengthen community engagement in policy implementation including through community-led monitoring. Participants recommended broader multi-stakeholder engagement that includes community and community health worker representatives in the development and implementation phases of new TB policies.

**Conclusions:**

Communities affected by TB, with the support of civil society organisations, could play a bigger role in monitoring policy implementation at local level and need to be capacitated to do this. This bottom-up approach could complement existing top-down strategies and contribute to greater accountability for TB policy implementation.

**Supplementary Information:**

The online version contains supplementary material available at 10.1186/s44263-024-00077-y.

## Background

Tuberculosis (TB) remains the leading cause of death in South Africa and in 2021 accounted for 56,000 deaths, with a further 304,000 people falling ill with TB [1]. A collaborative multisectoral effort is needed to find people with TB disease who are not currently receiving care and to support those diagnosed with TB to complete treatment. To reduce the impact this treatable and preventable illness has on society, one area that has not received sufficient focus is how accountability measures for TB policy implementation could contribute to these goals.

In the South African context, there are well-developed mechanisms for producing evidence-based policies by the TB directorate in the National Department of Health. This is supported by the TB Think Tank, a national network of TB experts. Existing South African TB policies scored highly in a multi-country audit led by the Stop TB Partnership and Médecins Sans Frontières in 2020 [2]. However, poor implementation hampers the impact of these policies. They have to transition through the provincial structures responsible for implementation, to district-level health services, and finally to frontline service delivery settings including hospitals, clinics, or community health centres [3, 4]. The challenges facing healthcare staff as implementers include the competing demands of clinical care for many other health conditions, policies developed by different departments that could contain contradictory guidance, resistance from staff or patients, misunderstanding the policy intent or recommendations, and resource challenges [3].

In 2022, the South African TB Recovery Plan was released to mitigate the detrimental effects of the COVID-19 pandemic on TB care, including a 41% decline in TB case notifications and a 26% decline in the number of people diagnosed with drug-resistant TB in 2020, compared to 2019 [5]. The underlying reasons for this include reductions in the number of diagnostic tests done and limitations in the health system’s capacity to provide TB services as well as decreased ability of people with TB to seek care in the context of restrictions on movement, concerns about transmission of COVID-19 at health facilities, and stigma due to overlapping symptoms between TB and COVID-19 [1]. The aim of the TB Recovery Plan was to produce a “target-driven, evidence-based plan aimed at finding people with undiagnosed TB, strengthening the linkage of people diagnosed with TB to treatment, retention in TB care, and TB prevention” [6]. Its core components included policies on expanding testing for TB (referred to as targeted universal testing for TB or the TUTT approach [7]), using new tools like digital chest radiography and urine lipoarabinomannan (LAM) tests for TB, ensuring that people diagnosed with TB are linked to care, and rolling out a more comprehensive TB Preventive Treatment programme to the close contacts of people diagnosed with TB to reduce their TB disease risk. The TB Recovery Plan was also a bridge to the new national strategic plan (NSP) for human immunodeficiency virus (HIV), TB, and sexually transmitted infections that was launched in 2023 and will be in place until 2028 and a response to the United Nations High-Level Meeting commitments on TB [8]. The National Department of Health TB directorate requested all nine provinces to develop provincial TB recovery plans. A focus area for this study is the Western Cape province which released a “Multi-sectoral Emergency Plan for TB Recovery”, developed in 2020 that aligned with the national plan that was in draft at that stage and aimed to define local targets on monitoring progress [9].

Theoretical approaches to understand policy implementation has generated two contrasting ideas: a top-down approach that emphasises command and control style leadership, places central (often national) policymakers as the key role-players, and emphasises developing clear policies with implementation being viewed as an administrative process that cascades down [3, 10]. In contrast to this, a bottom-up approach considers the power of the frontline implementers and is shaped by their ideas and interests. It considers how they may use local networks of service providers to either resist or support policy implementation [10]. A bottom-up approach to policy implementation provides greater autonomy to local implementers to determine how the policy is translated to their context and could generate more ‘buy-in’. A risk of the bottom-up approach is that it may lead to differentiated and unequal provision of care [10]. These two approaches can also be combined, to allow different levels of policy stakeholders to interact and collaborate on implementation, which requires close collaboration between policymakers and implementers [10]. The example of decentralised drug-resistant TB care policy implementation in South Africa demonstrates elements of a top-down approach, through a national directive to implement a new model of TB care, and a bottom-up approach, where frontline implementers were able to motivate for and also adjust and influence how the policy was implemented in practice. Le Roux and Kielmann described the implementation of this policy through focussing on the role of policy champions as a key resource for policy implementation, particularly at service delivery level, where they can help overcome resistance to implementation [11, 12].

Community-led responses and civil society engagement can make important contributions to ensure policies are responsive to the needs of people affected by TB and could also form part of bottom-up implementation strategies. Yet, the perspectives of civil society organisations (CSOs) and TB-affected communities on accountability for TB policy implementation, and how they may contribute to bottom-up implementation, are not well articulated in the academic literature. These two groups have been identified as important stakeholders in the World Health Organization (WHO) multi-sector accountability framework that focusses on strengthening TB policy implementation and have historically played an important role in advocating for HIV health policies in South Africa [13, 14]. There are several ways to engage communities in health policy—in priority setting, resource allocation, service management, implementation, and evaluation [15]. The mechanisms through which CSOs and TB-affected communities can strengthen accountability have not been well developed, and existing research has focussed on contributions of health committees linked to health facilities to influence local provision of care [16].

Using a qualitative approach, we aimed to (1) explore the implementation of the TB Recovery Plan in South Africa from provincial and local health department representative, civil society, and community perspectives and (2) to develop recommendations for strengthening accountability for policy implementation for this and future TB policies.

## Methods

We conducted 19 in-depth interviews and two group interviews (with two and three participants respectively) with a total of 24 stakeholders involved in or affected by TB policy development and implementation. We purposively sampled key informants from different stakeholder groups, including policy makers, policy implementers, health managers, community health workers (CHWs), civil society leaders, and community leaders. Participants were approached using email or telephone messages through existing professional networks. For different provincial perspectives, we included representatives who work across three high TB burden provinces (Western Cape, KwaZulu Natal, and Eastern Cape). For local implementers and community representatives, we focussed on the City of Cape Town area, including Khayelitsha, where TB Proof has existing relationships built through advocacy activities. We focussed on the Western Cape, which at the time of the interviews was the only province that had released a publicly available provincial implementation plan to accompany the national policy. We used snowball sampling by asking for recommendations for additional participants from the initial group aiming to reach thematic saturation. Interviews were conducted online using Zoom software (developed by Zoom video communications, California, version 5.15.12) or in-person, depending on the preference and location of the participant. Interviews lasted between 40 to 80 min and were conducted between August 2022 and October 2023.

Interviews were conducted by health workers IS or JG in English or TB survivors GM or PT in isiXhosa (for the five CHWs and four community representatives). They were audio recorded, transcribed, and the isiXhosa interviews were translated by an experienced research transcriber (SB). All finalised transcripts were reviewed for accuracy by the investigators who undertook the interviews (IS, JG, GM, or PT). All interviewers have experience in conducting qualitative research interviews and held team meetings to reflect on preliminary insights from the interviews and fieldnotes. Three of the interviews were female; one was male. The interview guide started with personal introductions and the goals of the study and then included questions on the content of the TB Recovery Plan, the contributions of policy to improving TB care in South Africa, and recommendations for implementing policy and accountability mechanisms (see Additional file 1). JG led the line-by-line data analysis, reviewing all transcripts and developing key themes which were then discussed by the wider team. Our approach to data analysis was informed by reflexive thematic analysis, which involved developing cross-cutting themes across the stakeholder interviews [17]. We used participant numbers and removed identifying information from the participant quotes. This paper adheres to the Consolidated criteria for reporting qualitative research (COREQ)—see Additional file 2.

## Results

For a summary of participants included in the study, see Table 1.

**Table 1:** Summary of participants

Stakeholder group	Number of participants	Additional notes
National and Provincial Department of Health representatives	8	Two participants were from the National Department of Health and six from the Provincial Department of Health. The provincial participants were from three provinces: Western Cape (*n* = 2), Eastern Cape (*n* = 1), and KwaZulu Natal (*n* = 3)
Local health structure representatives	6	This included district level and City of Cape Town health managers (Western Cape province)
Civil society organisational representatives	3	Organisational representatives involved in TB advocacy at local and national level (multiple provinces)
Community health workers	5	Khayelitsha (Western Cape province)
Community representatives	4	These included community representatives playing prominent religious roles (*n* = 2) a traditional leader (*n* = 1) and one worked in school food schemes (*n* = 1). Khayelitsha (Western Cape province)
Total	24	

We developed three themes regarding the implementation of the TB Recovery Plan in South Africa: the potential for COVID-19 innovation and urgency to influence TB policy development and implementation (theme 1), participant recommendations for closing the TB policy-implementation gap (theme 2), and strengthening the engagement of communities in TB policy implementation (theme 3).

### Theme 1: Potential for COVID-19 innovation and urgency to influence TB Recovery Plan development and implementation

The TB Recovery Plan was released within the broader context of the COVID-19 pandemic, and many participants mentioned this as a model for what an engaged, effective response to an infectious disease threat could look like. When considering the National Department of Health’s response to the COVID-19 pandemic, participants described the sense of urgency associated with responding to COVID-19, which they felt was lacking for TB. The global response to the COVID-19 pandemic and the sense that anyone can be affected was central to the momentum, as one participant pointed out:

“People cared about COVID because their families were dying. No one is going to care about TB until it starts actually affecting them.” – participant 2, local health structure.

Stakeholders reflected on the extensive resources dedicated to COVID-19, both globally as well as in South Africa. They also described the health system costs of focussing attention on a single disease. TB resources such as GeneXpert machines (a molecular rapid diagnostic test platform) were redirected to the COVID-19 response, and this led to weakening of the TB response as well as negative impacts on other priority health programmes. A participant said,

“We need to put the money where our mouths are. We can’t just talk about making things [referring to TB] a priority … and talk about ‘Oh this is an emergency’… but you not giving the resources that are required to really elevate the response.”—participant 9, Provincial Department of Health.

Participants drew parallels between the devastation caused by TB (viewed as very serious cumulatively) and COVID-19 (an acute emergency) but described how the TB programme was sidelined when COVID-19 became the priority.

The TB Recovery Plan was viewed as a positive response to focus more attention on TB, while incorporating innovations from the COVID-19 pandemic. For example, strategies for following up with patients and linking them to care through phone calls from dedicated call centres, and laboratories texting results, were now also recommended for TB. During the COVID-19 pandemic, data dashboards were used to share real time information on the epidemiology of the pandemic and use of resources, such as hospitalisation and use of oxygen, and were available to the public. The public-facing TB Dashboard in the Western Cape was an example of how this COVID-19 innovation was being piloted for TB.

“I think the TB Dashboard will help educate people around TB and it could help raise the profile of TB. If you begin to have such dashboards, you start to make them popular like what you manage to do with COVID dashboards. It’s a good initiative for everyone… for patients and for health care workers – it’s good for disease control.” – participant 11, National Department of Health.

Access to COVID-19 data in real-time also helped to highlight testing targets and compare performance across facilities. The TB Recovery Plan drew on this and placed a strong emphasis on data and monitoring outcomes across the TB care cascade. Civil society representatives mentioned that more needs to be done to make TB data available to CSOs and communities to drive accountability:

“For political buy-in… If you have the data, you can make them accountable – so collect the data and then make your case, make your demands to whoever is concerned, whether it’s a district or it’s a province…” – participant 10, civil society.

### Theme 2: Recommendation for closing the TB policy—implementation gap

Participants recommended four approaches to strengthen future TB policy implementation based on experiences with the TB Recovery Plan. Firstly, they recommended supporting TB champions in the health system. They noted that to advocate for TB policy implementation from within the national health system, senior TB programme officials have to navigate many obstacles that slow down document review and guideline release. This includes a complex regulatory environment, collaborations between different departments, and tender processes to purchase new drugs. High-level TB champions (for example National Department of Health representatives that are passionate about TB care) who are supported can usher a new policy through these processes so that its release is timeous and that urgency of implementation is communicated to provinces. Engaging a broad group of stakeholders can help raise awareness that the policy is in the pipeline, secure buy-in, including from civil society.

“We [civil society] had regular engagements, so I’m quite well aware of the different elements [to the TB Recovery Plan] and helped provide input into it.” – participant 15, civil society.

Participants described the development of the TB Recovery Plan as a participatory process, led by the National Department of Health, which helped to ensure more support for these high-level TB champions.

Secondly, participants drew attention to the importance of a policy being adequately resourced:

“The TB Recovery Plan is beautiful, really. But we need to know how it's going to be funded. Look at how fast COVID money came through and why can't the same be done to TB? You know, [what we need is] the political will.”—participant 14, civil society.

Inadequate resource allocation was also a barrier to provincial policy implementation, where local implementers felt they did not receive the resources they needed, including costed implementation plans. One participant summarised this as,

“Policies often stop at policy and don’t have implementation plans. When they do have implementation plans, they’re so complicated that nobody can work out how they work, and they don’t have realistic resource allocations. It’s critical to invest in the people, so they understand, and they do what they must do and to give them the tools to do what they must do, but keep it simple, and making sure that there is a resourced plan.”—participant 7, National Department of Health.

Only a minority of participants felt that there was adequate funding available for TB from local and international sources for core interventions. Several participants felt that it would be necessary to implement the TB Recovery Plan in a phased way and to indicate in which sequence the different parts of a policy should be prioritised.

For example, when considering implementing the targeted universal testing for TB (TUTT) component of the Recovery Plan (where people at high risk of TB are tested irrespective of whether they have TB symptoms), some policy implementers raised concerns about whether facilities would be able to manage the additional GeneXpert tests and the requirements for staffing, sputum booths, laboratory capacity, and supply of cartridges. In one province, rollout of this TUTT policy was partially underway, and they described this as being led by the province and not the National Department of Health. One participant emphasised the importance of communication:

“We at a provincial office must make sure that we communicate it clearly to the district. And then the district, sub-district—there must be a chain of communication. Once there is a gap somewhere, then there will always be poor implementation.”—participant 12, Provincial Department of Health.

They felt other helpful strategies were to engage with communities on the content of new policies, to ensure this was included in training manuals for all frontline staff and to convene multi-sectoral meetings prior to implementation.

Thirdly, participants felt that there should be stronger accountability measures in place at provincial level, to ensure that national policies are implemented at local level. Several participants mentioned delays and inefficiencies, and one participant highlighted that it was not always clear which mechanisms ensure that provincial health managers are held accountable for supporting implementation. There were also communication challenges in cascading new policies from provincial level to the frontline, including CHWs and community members, who are often not informed about new policies and therefore unable to contribute to community-led monitoring or demand generation for new TB services. One participant said:

“We still using outdated ways of communicating policies within the DoH. It’s via a circular, that’s sent from provincial office and there’s just then an assumption that somehow it’s going to feed down.” – participant 9, Provincial Department of Health.

Among our participants, the CHWs and community representatives were not aware of new TB policies, including the TB Recovery Plan. By comparison, the provincial-level participants and CSOs all knew about the plan, and many had participated in the policy development process. Provincial participants mentioned that CHW training is often fragmented which may contribute to them not being aware of new policies. In the Western Cape, that was because they are employed by NGOs, and in KwaZulu-Natal, their training is managed by a different provincial unit to the one that focusses on TB.

Fourthly, even though CHWs were widely recommended as key to the implementation of the TB Recovery Plan, and TB policies more broadly, it was also emphasised that supporting this cadre of health workers has not received sufficient attention and inclusion. Participants, including the CHWs, gave examples of how CHWs can assist with screening for TB through home visits, helping with retention in care as treatment supporters, and doing contact tracing for those who have been lost to follow up. One participant said,

“They are one of those critical enablers, part of that social mobilization of getting patients to the end of their treatment as well as getting contacts to the clinic, as well as doing testing out in the community.”—participant 5, local health structure.

Participant 2, based at a local health structure, described CHWs’ scope of work as “*horrifically wide”*—with recognition that the training, support, and supervision that they need is often lacking. Another participant said,

“The CHW is the most misunderstood and badly abused health care worker in our whole team, and yet, they are the foundation of what we should be doing.”—participant 7, National Department of Health.

CHW participants made explicit their concerns about the social determinants of health that affect TB patients, including food insecurity, conditions of desperate poverty, and poignant experiences of stigma. They described being impacted by the suffering they see:

“Some days you leave with a heavy heart because you heard their pain and the way they live.” – participant 20, CHW.

They felt that they could voice these priorities for TB patients in discussions about TB policy development and implementation and that this was not sufficiently addressed. One participant said,

“Most of the people do not work and they just sit at home without jobs, and they do not have food at home. The [TB] treatment makes people hungry. … I wish the government could provide food parcels for people with TB and make a grant for someone with TB.”—Participant 22, CHW.

Policymaker participants advised that empowering CHWs and formalising their role in the health system would make a significant impact on TB services as well as policy implementation. This includes improving CHW training, job descriptions, and supervision.

“I know that as much as we say they play a very important role, their contracts have not been really given the necessary attention that they should be getting.” – participant 11, National Department of Health.

The majority of the participants that we interviewed viewed CHWs as implementers of policy and not contributors to policy development.

### Theme 3: Strengthening engagement of communities in TB policy implementation

Participants mentioned that historically, in South Africa, health managers and providers have viewed communities as passive recipients of services. While there were some examples of a shift towards community consultation and co-creation, participants felt that national, provincial, and local health departments needed to engage more proactively with community representatives, instead of being defensive during engagements. As one participant noted,

“There’s still this idea that we as the DoH need to come with something and we will negotiate around it, but certain things will remain non-negotiable … and we will give a little bit here, but we are not really responding to the needs of the community. And you are actually just making people feel like you are using them and that it’s a tick box exercise … it’s a compliance exercise … and they become frustrated and then they don’t want to participate anymore.”—participant 9, Provincial Department of Health.

Community perspectives emphasised the need to improve knowledge and awareness about TB and noted that getting access to decision makers within government can be difficult, except for CSOs that had a strong national profile. One participant made a direct request:

“I believe that if we can receive in-service training or training on recovery plans, we will be able to be aware of it and talk about it in our spaces, such as in our communities and families, as well as traditional ceremonies. … There is a gap in our knowledge as traditional healers where we need further training in order to be able to advocate for TB.”—participant 16, community.

There was consensus from participants that communities need more engagement on TB policies, through information campaigns and a variety of media, with materials provided in local languages and distributed beyond health facilities.

Challenges for community members to access data, such as whether targets for policy implementation were being met, were raised. CSO participants described how organisations can collaborate closely with communities to promote community-led monitoring—for example engaging with communities on what TB services should be like, discussing common service provision challenges, and doing facility-level investigations to provide feedback. Examples were given of activities by a local NGO, Ritshidze, in clinics where community members audit clinics for key TB policy indicators, such as whether the urine LAM diagnostic test was available or assessing turnaround time for GeneXpert results. This approach also incorporates data that is used to compile health service delivery reports for specific districts. Participants viewed access to local data as central for all stakeholders in the TB response to improve accountability.

One participant suggested broader use of KwaZulu Natal province’s ‘war rooms’ which provide a meeting point for community engagement with government structures:

“[This is] where community representatives and government departments sit and discuss challenges in a particular community: health related, development related, or anything that worries the community. In those forums, basic data and TB related information can be presented.”—participant 1, Provincial Department of Health.

Health committees (where community representations serve on an official structure linked to a health facility) were mentioned as an existing mechanism within the South African health system where communities can potentially contribute to accountability and that this could be used to track TB policy implementation. Participants highlighted that these committees should include community-based CSOs and community leaders and must be diverse, representative, informed, and empowered. Capacitation is needed to ensure that community members are not simply incorporated into the health facility and that they fulfil their roles as independent observers. Supporting community representatives in their role as health committee members was an area where participants felt CSOs can help drive accountability.

“We need to create a context where everybody knows that they have a role to play, and they can’t just sit and wait for the Health Department or the TB programme to take action.”—participant 11, National Department of Health.

Participants also felt that greater CSO representation, at all levels of the health system, can help to voice community priorities at the different levels of policy development and implementation.

“Civil society [need] representation at all levels. From the clinic committee to the hospital board, to the District Health Council, to the Provincial Health council and to the National Health Council so that we can represent the community and have an understanding on how these decisions are made.” – Participant 10, civil society.

To summarise our key findings from themes 1 to 3, the following key messages presents various ways in which learning from the implementation of the TB Recovery Plan can be translated to different TB policies, including implementation of the new NSP.

Key messages from participants to support future TB policy implementation:

- Current participatory approaches to policy development strengthen national, provincial, and civil society awareness of policies, but targeted efforts are needed to better engage local-level frontline workers and communities.
- It is important to identify and support champions for TB policies in all levels of the health system.
- Implementation of the TB Recovery Plan and the NSP needs to be adequately resourced, which could be addressed through developing costed provincial implementation plans.
- Greater emphasis needs to be placed on accountability measures to ensure translation takes place from national to provincial policy, to local implementation.
- To strengthen community-level engagement in policy implementation, policymakers need to value the contributions of CHWs and community members, who should be capacitated to engage and provide input.
- CSOs can help facilitate engagement between policymakers and TB affected communities and play a key role in capacitating communities to engage in policy implementation.
- Strengthening community awareness, through improved public communication about TB and TB policies, can engage a much wider audience in the national TB response, build a stronger demand for quality services, and increase accountability for policy implementation.

## Discussion

Our multistakeholder analyses identified three themes that provide critical insights for TB policy implementation. Firstly, participants reflected on how the COVID-19 pandemic created a sense of urgency to catch up on TB progress that had been lost and highlighted innovations that could be incorporated into TB policy. Secondly, they recommended supporting TB champions across health system levels, adequate resourcing for new TB policies, stronger provincial accountability mechanisms, and more support for the role of CHWs and local-level partners to reduce the policy-implementation gap. Thirdly, participants recommended that to enhance accountability for TB policy implementation at community level, more community members need to be informed on why TB is an important topic in South Africa and what a new policy aims to do.

When interpreting our findings using the top-down and bottom-up approach to policy implementation, we found that with the TB Recovery Plan, the top-down approach was dominant [10]. There were mechanisms for input on the policy from different levels, with national and provincial policymakers engaging in the process, although there was a perceived lack of TB champions to advance this policy and prioritise its implementation. There was a major bottleneck at provincial level, whereby participants felt there were limited accountability measures in place to ensure the national policy is embraced by provincial-level policymakers and championed for implementation. This is not unique to TB policies; similar concerns have been documented for mental health policy implementation in South Africa [18]. Provinces face severe human resource and infrastructure constraints that hamper policy implementation, and attempts to resolve this bottleneck should avoid punitive monitoring mechanisms where a focus on compliance detracts from processes that can improve quality of care [19].

In the Western Cape, we found that CHWs and community representatives were not aware of the TB Recovery Plan and were not engaged in bottom-up approaches to inform how TB policy translates to implementation at a local level. While the lack of CHW awareness and engagement in the Western Cape may reflect the employment landscape in this province, which is NGO-based, similar concerns were raised by a national-level policymaker and we note the challenges of ensuring high quality TB training for CHWs given their wide scope of work. Notably, KwaZulu Natal province has started to engage communities in their TUTT policy release; however, our dataset does not allow direct comparison with how this impacted implementation. We hypothesise that having fewer frontline health worker and community champions for the TB Recovery Plan overall, when compared to HIV and drug-resistant TB policies in South Africa, has led to less demand for implementation. Lessons from HIV and drug-resistant TB policy implementation include having CSOs as champions that advocate for local implementation and that engage communities in new policies being released [11, 12, 14]. An example is the treatment literacy campaigns for people living with HIV that the Treatment Action Campaign ran, which stimulated demand and emphasised the critical implications of policy implementation for people living with HIV [14]. To ensure a strong bottom-up approach for the TB Recovery Plan, input from frontline health workers on resourcing requirements and other implementation barriers should be included, alongside community collaborations at local level to advocate for policy implementation.

Community-led monitoring of policy implementation, through the use of health facility audits or public data dashboards, would help support a bottom-up approach [13]. To enable this, communities need to be engaged more broadly with TB as a public health priority and on new policies being implemented. This should be expanded to include input into policy development. CSOs can play a key role in capacitating communities at local level and representing these perspectives in provincial and national meetings [14]. To do so, CSOs need resources, independent of the National Department of Health, to fulfil this accountability role.

This proposed accountability role for communities and CSOs aligns with the WHO Multisectoral Accountability Framework for TB that recommends civil society and community input on periodic national policy reviews, strategic plans, and revision of these plans based on monitoring [13]. As this role is explored further in the South African context, future research should document best practice examples for the use of this WHO Framework. The TB Accountability Consortium, led by the Rural Health Advocacy Project, is one example of a civil society platform that aims to explore this role [20]. We suggest combining a bottom-up and top-down approach to TB policy development and implementation, because by broadening the stakeholders involved in developing policy, it could strengthen the implementation of policies such as the new NSP.

Strengths of this study include engagement of a diverse range of stakeholders enabling inclusion of important perspectives from actors less commonly engaged in TB policy implementation, and the interdisciplinary skillset of the research team, which spans expertise in public health, community engagement, TB advocacy, personal experience of TB, and TB research. Limitations of our study include that the frontline health worker perspectives were limited to CHWs and one doctor and that our local health structure representatives and community perspectives were from participants based in the Western Cape province.

## Conclusions

Multistakeholder perspectives on the implementation of the TB Recovery Plan in South Africa emphasise that accountability at provincial level is an important bottleneck in the process of policy implementation. Key recommendations include the use of provincial-level TB data dashboards to facilitate iterative improvements based on performance feedback and engaging CSOs in tracking provincial and local policy implementation. Communities affected by TB, with the support of CSOs, should be capacitated and supported such that they can play a valuable role in monitoring policy implementation at local level.

## Supplementary Information

Additional file 1: Interview guides

Additional file 2: COREQ Checklist

## Data Availability

The datasets generated and/or analysed during the current study are not publicly available due to ethics approvals requiring us to protect the confidentiality of our participants. Through full transcriptions, participants may be identifiable through their prominent positions in the South African policy landscape. To discuss access to anonymised segments and any further requests for the data further, please contact the corresponding author at helene.vdw@phc.ox.ac.uk.

## Abbreviations

CHW: Community health worker
COVID-19: Coronavirus disease 2019
CSO: Civil Society Organisation
DoH: Department of Health
HIV: Human immunodeficiency virus
NSP: National Strategic Plan for HIV, TB and Sexually Transmitted Infections
TB: Tuberculosis
WHO: World Health Organisation
