# Supplementary material for: Strengthening accountability for tuberculosis policy implementation in South Africa: perspectives from policymakers, civil society, and communities
Source: BMC Glob Public Health. 2024 Jul 17;2:48. doi: 10.1186/s44263-024-00077-y (PMC11252195; doi:10.1186/s44263-024-00077-y)
Supplement: Supplementary file 1 — Additional file 1: Interview guides [file 44263_2024_77_MOESM1_ESM.docx]

**Additional file 1: Interview guides**

**Interview guide: Policy-level decision makers and health workers**

**UNIVERSITY OF THE WESTERN CAPE**
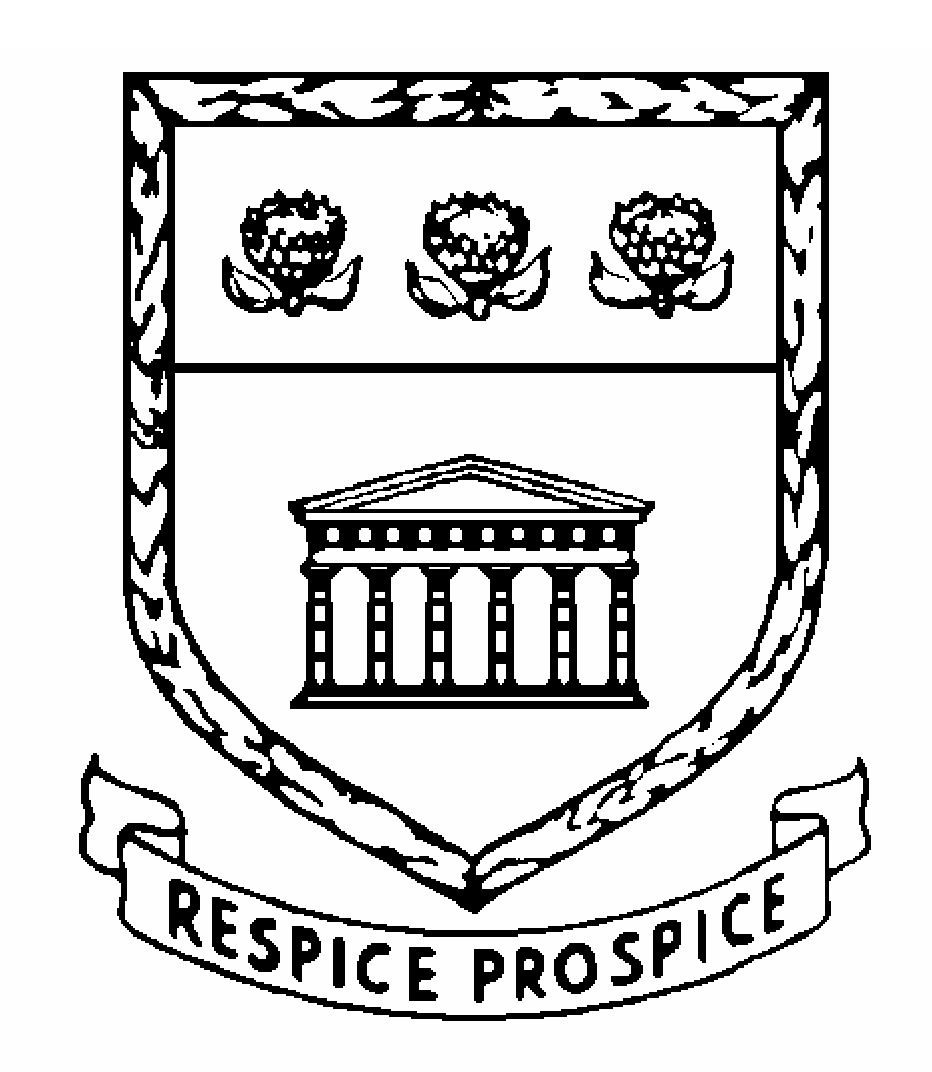


## **Private Bag X 17, Bellville 7535, South Africa**

***Tel : +27 21-959 2632***  **E-mail :** [**recoetzee@uwc.ac.za**](mailto:recoetzee@uwc.ac.za)

**Introduction of the interviewer**

Thank you for agreeing to take part in this study. This interview is for a research study to understand your knowledge and experience of the TB Recovery Plan. We greatly value your perspective. There are no right or wrong answers to the questions we will ask.

I would like to digitally audio record our conversation using this device through your consent. This will allow us to type our conversation, but any organisation names or places that you mention will be taken out so that you will be anonymous. This way, if someone were to read this interview, they would not be able to know who you are because your organisation will be replaced with a code. If at any point during the interview you do not wish to answer a question or stop participating in the study, that is okay.

Do you have any questions?

**Topics**

*Below is a list of topics that can be discussed during the in-depth interviews. The questions will remain flexible to the participants’ agendas, and new topics may be added as the interviews progress. The key topic of the implementation of the TB Recovery Plan will remain central throughout the interview.*

*Please note that this interview guide will be refined based on feedback from stakeholder discussions. The following are examples of questions that could be used. Given this study’s methodology (semi-structured interview), additional questions may be added during interviews following the topics listed above.*

**Topic Area 1 - Personal introduction**

Please tell me more about your organisation/ department and what it does.

Please tell me about your role and time of service in your organisation/ department and how this relates to TB.

Prompts: Could you share with me why you care/ are passionate about TB?

What area of health is your organisation focused on? (E.g. maternal health etc)

In which areas in SA are projects implemented? (Prompts: Can ask specifically about Khayelitsha and Hammanskraal)

**Topic Area 2 - Knowledge about the TB Recovery Plan and CHWs’ roles in the TB Recovery Plan**

Are you aware of the TB Recovery Plan released this year? Prompt: Were you part of any meetings or platforms where you provided input regarding its content?

The TB Recovery Plan outlines key strategies to prevent TB, reach people with TB, link them to care, and support them on treatment to help close gaps in the TB care cascade. Could you please describe, based on your experience, which three key interventions you think should be implemented as priorities?

Based on your experience, where are major service delivery gaps that could be addressed to implement TB policies better at community-level? Prompt: what are your recommendations to decrease gaps between policy release and implementation.

How do you think community members could keep the Department of Health accountable to implement TB policies or support policy implementation?

Could you please tell us more about your department’s/ organisation’s interaction with community health workers (CHWs) in the area you work? Please describe their roles in the TB response?

If your organisation employs CHWs, what training on TB policies are available to them? (Prompts: How long is the training, by whom, how regular, any refresher training).

What do you think is important for TB training for CHWs to cover?

How do you think that CHWs could be better involved and supported in the TB response?

**Topic Area 3 - Questions about TB**

How does your organisation support TB services? Prompt: Does your organisation target any specific groups of people who might be at high risk of getting TB?

What sort of information campaigns regarding TB are you familiar with in your catchment area? [Could prompt to ask about campaigns they felt were successful, contextually relevant e.g. local languages or innovative.]

Does your department / organisation publish TB data/ statistics? [If yes] What TB statistics does your organisation share, with whom and on which platforms?

Do you think TB is given enough priority by your organisation? (Prompt – could elaborate.)

**Topic area 4 - Access to TB testing and care**

What types of outreach events for TB testing are happening in your community?

Which TB tests does your organisation offer, if any?

Based on your experience, which people in the community struggle to access TB care? What are the main barriers? What changes can be implemented to ensure that more people can have access to TB testing and care?

Can you elaborate on the counseling being provided to people diagnosed with TB? [e.g. does counseling include screening for mental health challenges issues such as depression or for alcohol and substance use with appropriate follow up where necessary to a psychologist, social worker or dietitian?]

How are people diagnosed with TB linked to a TB support group and informed about the HIV helpline if they need any support (even if they don’t have HIV)?

Could discuss specific components of TB recovery plan and their implementation, eg.:

How can awareness campaigns among high-risk asymptomatic groups such as people living with HIV help to increase TB testing?

Are you aware of TB Preventive Therapy and that new guidelines were released this year?

What can be done to ensure that TB contacts and high risk groups such as PLHIV access TB Preventive Therapy (TPT)? What are barriers to accessing TPT?

Putting that all together, how do you think we can reach more stakeholders to support TB services?

Thank you very much for your time, we are doing this because we want to find ways to support TB policy implementation in South Africa

Could provide summary of interview and ask whether participants have any additional insights to share or adaptations to suggest.

#

**Interview guide: Community Health Worker**

**UNIVERSITY OF THE WESTERN CAPE**
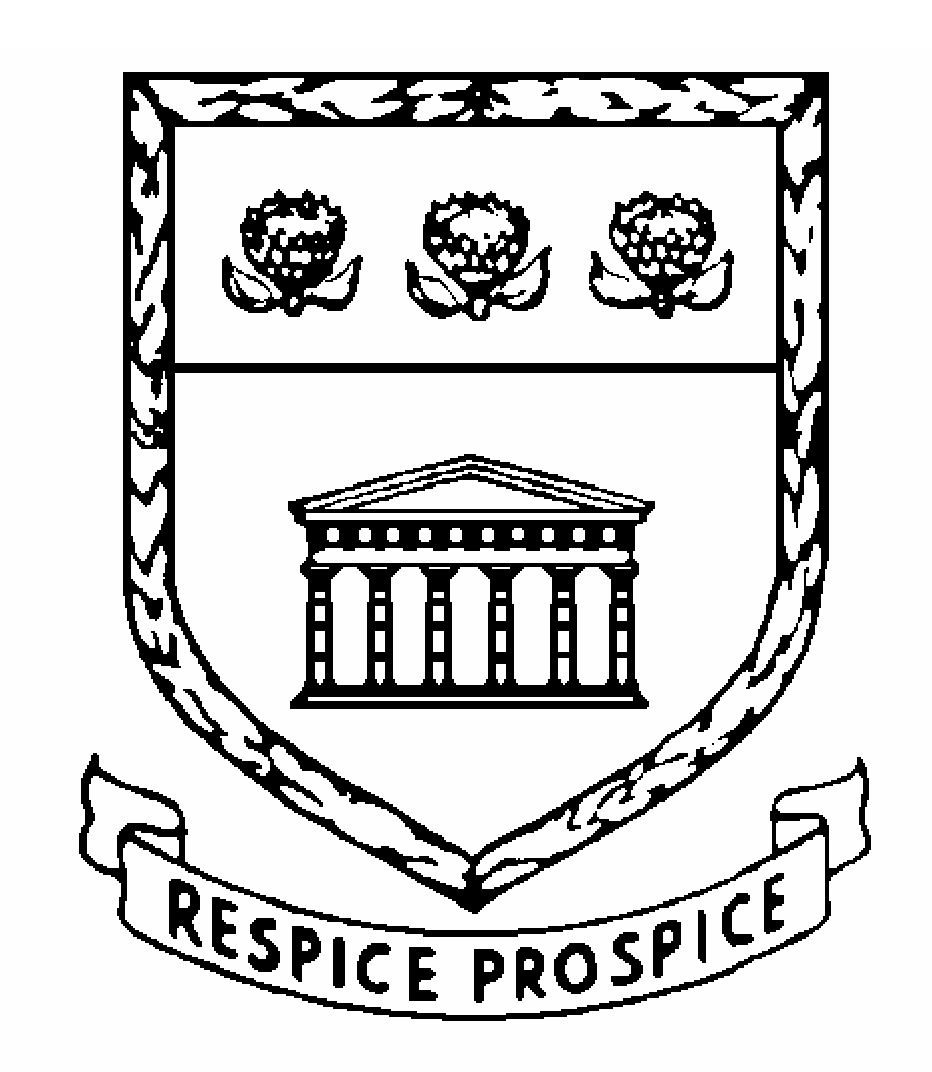


## **Private Bag X 17, Bellville 7535, South Africa**

***Tel : +27 21-959 2632***  **E-mail :** [**recoetzee@uwc.ac.za**](mailto:recoetzee@uwc.ac.za)

**Introduction of the interviewer**

Thank you for agreeing to take part in this study. This interview is for a research study to understand your experiences of caring for patients with TB and how you are involved with new TB policies. We greatly value your perspective. There are no right or wrong answers to the questions we will ask.

I would like to record our conversation using this recorder through your consent. We will also take some photos. This will allow us to type our conversation, but any names or places that you mention will be taken out so that you will be anonymous. This way, if someone were to read this interview, they would not be able to know who you are because your name will be replaced with a code. If at any point during the interview you do not wish to answer a question or stop participating in the study, that is okay.

Do you have any questions?

**Topic Area 1 - Personal introduction**

Can you tell me a bit about yourself:

How old are you? Why did you become a CHW? When did you start working as a CHW?

Tell me about your current role providing care for people with TB?

**Topic Area 2 - Questions about TB training**

Please tell me more about the training you received regarding TB?

Prompts: Please tell me more about the different TB topics that were covered? How was the training done and who delivered it? E.g. Did you receive once-off training on TB or is refresher training being done?

What additional training on TB would you like? (Prompt: Have you ever visited a household where they asked questions about TB that you did not know the answers to?)

How often do you think TB training should be done?

Who is providing you with support at work?

**Topic area 3 - Knowledge about TB**

When community members ask you about TB, how do you describe this disease? (Prompts: How does it spread? Can TB be prevented?)

What kind of protective equipment are you provided with at work (e.g. N95 respirators or surgical masks)?

Does everyone with TB have a cough, weight loss, fever or nights sweats, or have you met some people who do not show any symptoms?

Have anybody you have helped used the self-screening applications on a cellphone for TB like TB Check?

What TB tests are available for people who you want to investigate further?

What public TB statistics available on the internet are you aware of? (Similar to the COVID-19 statistics on television, but specifically for TB).

Following the COVID-19 pandemic the government wrote a plan to improve TB care. This document is called the TB Recovery Plan. Have you heard about it?

If you were to give advice to the government about how to improve TB care for people in your community, what would you recommend?

Prompts *(ask one question at a time):*

- How can we better reach people who might have TB in the community and link them to care?
- What is needed to support people on TB better to complete treatment?
- How can TB be prevented in our communities?

The government releases guidelines about how to manage all illnesses, including TB so that health workers at the facilities know what to do. For example, if there are new tests for TB or new drugs for TB the government shares the details in guidelines.

Have you had experiences where these types of guidelines are shared with you?

Prompts: do you have any suggestions on how the Department of Health should share such policies with CHWs? How would you like to receive this information?

**Topic area 4 - Recommendations about CHWs roles**

If you or another community health worker is visiting a household where someone is coughing, what services do you provide and recommend?

If you know of a patient diagnosed with pulmonary TB, what is the role of community health workers to provide care for other household members?

Thinking about TB compared to other diseases, how do you feel people react to it compared to high blood pressure or diabetes?

Based on your work with people who have TB, who do you think struggles to access TB care? (Prompts: What are the main barriers? What would make it easier for more people to get tested for TB?) Why do you think is it difficult for some people to complete treatment?

What support do you need in your role as community health worker to help people with TB better?

Thank you very much for your time, we are doing this because we want to find ways to support TB policy implementation in South Africa.

[Could provide summary of interview and ask whether participants have any additional insights to share or adaptations to suggest.]

Thank you.

**Interview guide: Community leader interview**

**UNIVERSITY OF THE WESTERN CAPE**
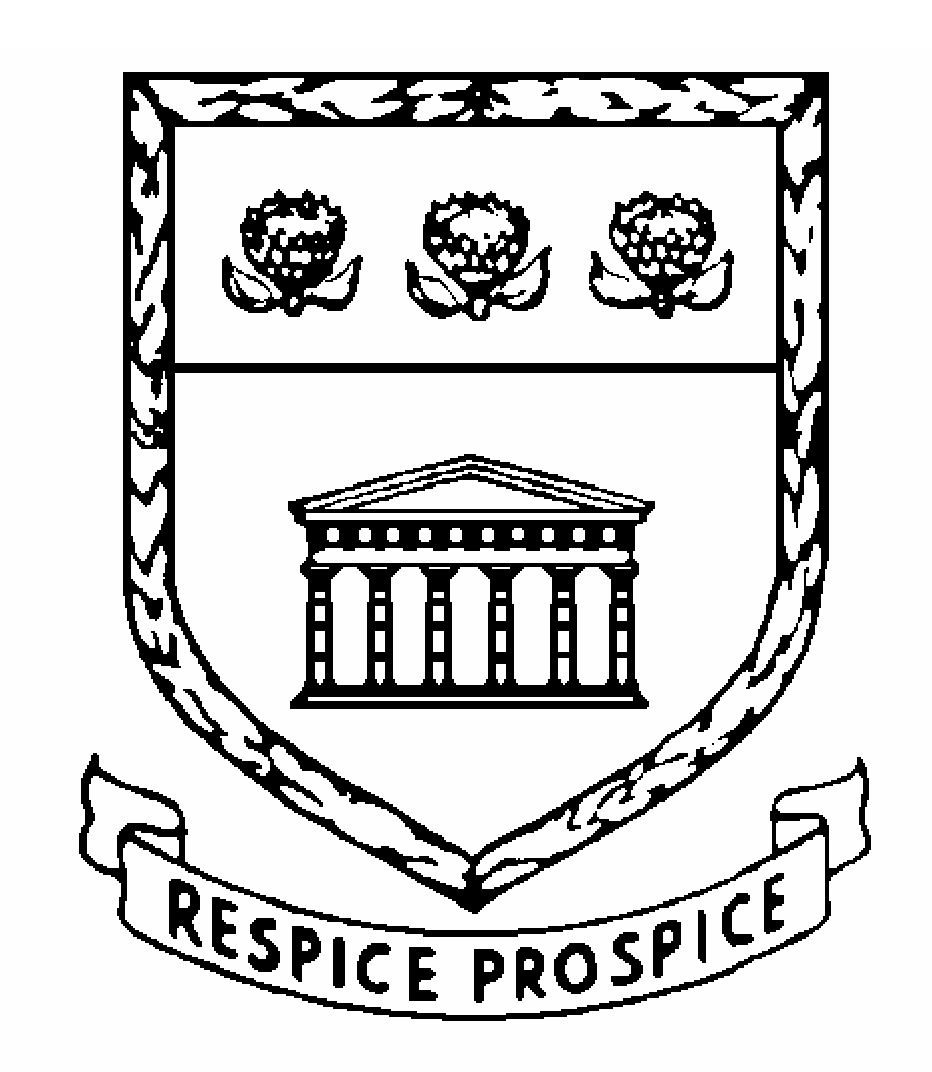


## **Private Bag X 17, Bellville 7535, South Africa**

***Tel : +27 21-959 2632***  **E-mail :** [**recoetzee@uwc.ac.za**](mailto:recoetzee@uwc.ac.za)

**Introduction of the interviewer**

Thank you for agreeing to take part in this study. This interview is for a research study to understand your perspectives as a community leader on TB and how you are involved with new TB policies. We greatly value your perspective. There are no right or wrong answers to the questions we will ask.

I would like to record our conversation using this recorder through your consent. We will also take some photos. This will allow us to type our conversation, but any names or places that you mention will be taken out so that you will be anonymous. This way, if someone were to read this interview, they would not be able to know who you are because your name will be replaced with a code. If at any point during the interview you do not wish to answer a question or stop participating in the study, that is okay.

Do you have any questions?

**Topic Area 1 - Personal introduction**

Can you tell me a bit about yourself and your role in the community?

If relevant: name of your organisation or department, time of service and link to TB services?

Could you please tell us more about your department’s/ organisation’s interaction with community health workers (CHWs) in the area you work?

**Topic Area 2 – Understanding of TB**

When community members ask you about TB, how would you describe this disease?

How does it spread?

Can TB be prevented?

In your experience, does everyone with TB have a cough, weight loss, fever or nights sweats, or do some people not show any symptoms?

Have anybody you have met used the self-screening applications on a cellphone for TB like TB Check?

What types of outreach events for TB testing are happening in your community? What do you think sets the successful events apart?

Do you think TB is treated with the same importance as other diseases e.g. diabetes or high blood pressure?

Based on your experience, which people in the community struggle to access TB care? What are the main barriers?

If you were to give advice to the government about how to improve TB care for people in your community, what would you recommend?

Prompts *(ask one question at a time):*

- How can we better reach people who might have TB in the community and link them to care?
- What is needed to support people on TB better to complete treatment?
- How can TB be prevented in our communities?

In your community context, are you aware of counseling being provided to people diagnosed with TB? Are there any TB support groups?

**Topic area 3 - Accountability**

The government releases guidelines about how to manage all illnesses, including TB so that health workers at the facilities know what to do. For example, if there are new tests for TB or new drugs for TB the government shares the details in guidelines.

Have you had experiences where these types of guidelines are shared with you?

Prompts: do you have any suggestions on how the Department of Health should share such policies with community leaders? How would you like to receive this information?

Have you heard of any such TB guidelines?

How can communities stand together to share their recommendations to improve TB care with the government? What would you recommend?

Thank you very much for your time, we are doing this because we want to find ways to support TB policy implementation in South Africa.

[Could provide summary of interview and ask whether participants have any additional insights to share or adaptations to suggest.]

Thank you.
